# Measures of Serum Markers HbA1c, HOMA-IR, HOMA-β, QUICKI and G/I Ratio as Predictors of Abnormal Glucose Tolerance Among Thai Women with Polycystic Ovary Syndrome

**DOI:** 10.3390/jcm14051452

**Published:** 2025-02-21

**Authors:** Thanyarat Wongwananuruk, Pichita Prasongvej, Panicha Chantrapanichkul, Suchada Indhavivadhana, Prasong Tanmahasamut, Manee Rattanachaiyanont, Kitirat Techatraisak, Surasak Angsuwathana

**Affiliations:** 1 Gynaecologic Endocrinology Unit, Department of Obstetrics and Gynaecology, Faculty of Medicine Siriraj Hospital, Mahidol University, Bangkok 10700, Thailand; 2 Department of Obstetrics and Gynaecology, Faculty of Medicine, Thammasat University, Pathum Thani 12120, Thailand

**Keywords:** serum markers, glucose intolerance, PCOS

## Abstract

**Background/Objectives**: Polycystic ovary syndrome (PCOS) is a prevalent endocrine disorder affecting women of reproductive age. It is associated with glucose intolerance, insulin resistance and diabetes mellitus. The oral glucose tolerance test (OGTT) is commonly employed to detect glucose intolerance, but it can be inconvenient and time-consuming. We aimed to evaluate the precision of haemoglobin A1c (HbA1c) and other serum markers in predicting abnormal glucose tolerance (AGT). **Methods**: This diagnostic study involved 121 PCOS women who attended the Gynaecologic Endocrinology Unit at Siriraj Hospital. Patients underwent assessments for weight, height, waist circumference, modified Ferriman–Gallwey score and acanthosis nigricans. Blood samples were collected to measure fasting glucose and insulin levels after a 75-gram oral OGTT, fasting insulin level, HbA1c, lipid profile, androgen profile and complete blood count. Homeostasis model assessment of insulin resistance (HOMA-IR), homeostasis model assessment of beta-cell function (HOMA-β), quantitative insulin sensitivity check index (QUICKI) and fasting glucose-to-insulin ratio (G/I ratio) were calculated. The sensitivity, specificity and accuracy of these serum markers were compared. **Results**: The prevalence of AGT was 24.8%. The area under the receiver operating characteristic curve for HbA1c in detecting AGT was 0.656, while HOMA-IR, HOMA-β, QUICKI and the G/I ratio had values of 0.817, 0.737, 0.817 and 0.77, respectively. The G/I ratio cut-off point of 6% demonstrated a sensitivity of 73.3%, specificity of 74.7%, positive predictive value of 48.9%, negative predictive value of 89.5% and accuracy of 74.4%. **Conclusions**: The G/I ratio is the most accurate compared to other serum markers in detecting AGT among Thai women with PCOS.

## 1. Introduction

Polycystic ovary syndrome (PCOS) is a prevalent endocrine disorder affecting women of reproductive age, with a prevalence ranging from 8% to 13%, depending on the studied population and the utilised definition [1,2]. The Revised Rotterdam 2003 criteria are commonly employed for diagnosing PCOS [3]. This syndrome manifests with menstrual irregularity, hyperandrogenaemia or hyperandrogenism, or polycystic ovaries [4]. Women with PCOS demonstrate a higher prevalence of impaired glucose tolerance, insulin resistance and diabetes mellitus (DM) than women without PCOS [5,6].

For diagnosing glucose intolerance in PCOS women, the Rotterdam ESHRE/ASRM-sponsored PCOS consensus workshop group recommends using a 75-gram oral glucose tolerance test (75 g OGTT) [3]. The use of only fasting glucose underestimates glucose intolerance [7]. The Androgen Excess and PCOS Society 2007 guidelines recommended screening all PCOS women using a 2-hour 75 g OGTT [8]. However, the 2010 guidelines suggest employing the 2-hour 75 g OGTT only in obese individuals (BMI > 30) or lean or overweight individuals with at least one additional risk factor, such as a history of gestational diabetes mellitus, family history of DM or advanced age [9]. Recent recommendations propose evaluating the baseline glycaemic status, fasting plasma glucose or haemoglobin A1c (HbA1c) in all women with PCOS [10]. Despite its diagnostic utility, the OGTT can be inconvenient and time-consuming, requiring patients to fast and undergo blood collection twice.

Haemoglobin A1c (HbA1c) is a form of haemoglobin A that incorporates a glucose molecule into the N-terminal valine of the beta chain. It serves as a marker of chronic glycemia, reflecting the average plasma glucose level over 2 to 3 months. HbA1c exhibits no day-to-day variation, requires no fasting before testing and involves only a single blood sample, making it convenient and time-efficient [11]. The American Diabetes Association recommends HbA1c and fasting glucose as alternatives to the 75 g OGTT for screening for type 2 DM and prediabetes [12]. Therefore, HbA1c may potentially be usable instead of the OGTT to detect glucose intolerance.

Numerous studies have investigated serum markers for predicting abnormal glucose tolerance (AGT) in PCOS women. Other tests used to assess glucose intolerance include homeostasis model assessment of insulin resistance (HOMA-IR), homeostasis model assessment of beta-cell function (HOMA-β), quantitative insulin sensitivity check index (QUICKI) and fasting glucose-to-insulin ratio (G/I ratio). These markers offer the advantage of not requiring complex laboratory tests. The methods use different equations to evaluate fasting plasma insulin and fasting plasma glucose levels. Studies conducted in other countries have examined the use of HOMA, QUICKI and the G/I ratio to assess glucose levels in PCOS women [13,14,15,16].

Despite the recent reporting of various serum markers for predicting AGT in PCOS women, data on their effectiveness remain limited. This study evaluated the accuracy of different markers in detecting AGT among Thai PCOS women and compared them to other tests.

## 2. Materials and Methods

We announced this research by posters attached to the hospital and provided information from nurses in the Gynaecologic Endocrinology Unit when patients were diagnosed with PCOS. One hundred and twenty-one PCOS women, aged 18 to 45 years, were recruited from patients attending the Gynaecologic Endocrinology Unit, Department of Obstetrics and Gynaecology, Faculty of Medicine Siriraj Hospital, between May 2019 and January 2020. Their diagnoses of PCOS were made according to the revised Rotterdam criterion of 2003 [3]. This study protocol was reviewed and approved by Siriraj Institutional Review Board, approval number Si-255/2019.

Exclusion criteria encompassed women using hormonal contraception or steroids during the previous 3 months and those taking insulin-sensitising, lipid-lowering drugs or other medications influencing insulin sensitivity within the last 6 months. Pregnant and breastfeeding women were also excluded.

The medical histories of all participating PCOS women were recorded, and comprehensive physical examinations were conducted. Measurements of height, weight, blood pressure and waist circumference were obtained. The modified Ferriman–Gallwey score was assessed, and the presence of acanthosis nigricans was documented. Body mass index (BMI) was calculated and classified into four groups per the World Health Organisation criteria: underweight (<18.5), normal (18.5–22.9), overweight (23–24.9) and obese (≥25) [17].

Total venous blood sampled was about 24–36 mL. Venous blood samples were collected twice. After an overnight fast (at least eight-hour fast), the first collection was performed between 8:00 a.m. and 10:00 a.m. This sample included measurements of fasting plasma glucose, HbA1c, sex hormone-binding globulin, dehydroepiandrosterone sulphate, testosterone, albumin and a complete blood count. The second blood sample was drawn after a 2-h oral glucose ingestion of 75 g. Free testosterone was calculated using a specific calculator [18].

Laboratory assays were conducted at the laboratory unit of the Department of Clinical Pathology, Faculty of Medicine Siriraj Hospital, Mahidol University. This laboratory holds ISO 15189 [19] accreditation, ensuring adherence to rigorous quality standards. All assays were performed using an automated analyser. The coefficients of variation for intra- and inter-assay measurements were below 5% for all techniques.

HbA1c analysis was conducted using a Cobas c513 analyser from Roche (Basel, Switzerland). Additionally, sex hormone-binding globulin, dehydroepiandrosterone sulphate, testosterone and insulin were assessed using electrochemiluminescence immunoassay on a Cobas 8000 modular analyser (Roche, Basel, Switzerland). Glucose measurements were performed using the enzymatic hexokinase method on the Cobas 8000 modular analyser. Complete blood count analysis was performed using electrical resistance, hydrodynamic focusing and the sodium lauryl sulphate–haemoglobin method on a Sysmex XN-3000 automated haematology analyser (Sysmex Corporation, Kobe, Japan).

Any of the following factors indicated AGT: impaired fasting glucose, impaired glucose tolerance test and DM. ‘Impaired fasting glucose’ was defined as fasting glucose levels ≥ 100 and <126 mg/dL. ‘Impaired glucose tolerance’ was defined as 2-h plasma glucose levels ≥ 140 and <200 mg/dL, while ‘DM’ was defined as fasting glucose levels ≥ 126 mg/dL or 2-hour plasma glucose levels ≥ 200 mg/dL [12].

This study used several additional methods to detect glucose intolerance: HOMA-IR, HOMA-β, QUICKI and the fasting G/I ratio. HOMA-IR was calculated by multiplying fasting plasma glucose (mg/dL) by fasting plasma insulin (µU/mL) and dividing the result by 405. HOMA-β was calculated using the formula (360 × fasting plasma insulin [µU/mL])/(fasting plasma glucose [mg/dL] − 63) [20]. QUICKI was calculated as 1 divided by the logarithm of fasting plasma insulin (µU/mL) plus the logarithm of fasting plasma glucose (mg/dL) [21]. The G/I ratio was calculated by dividing fasting plasma glucose (mg/dL) by fasting plasma insulin (µU/mL) [13].

The sample size calculation was based on the findings of a study by Celik et al. Their investigation established a cut-off level for HbA1c of 5.35% for detecting AGT (sensitivity: 75.6%; specificity: 52.6%) [22]. Drawing on this information, it was estimated that 22 PCOS women with AGT would be needed for our study. As the current study was cross-sectional, the prevalence of AGT in Thai PCOS women was assumed to be 20% based on a study by Wongwananuruk et al. [23]. After including an additional 10% of subjects to account for incomplete data, the total sample size was 121 cases.

Normality testing was done using the Kolmogorov–Smirnov test. Normal and non-normal distributions are presented as mean and median with percentiles, respectively. Descriptive data are presented as mean ± SD for continuous variables and median with percentiles at the 25th and 75th percentiles. Percentages are provided for categorical data. Statistical analyses included the Mann–Whitney U-test, chi-squared test and *t*-test for comparisons between two groups. Receiver operating characteristic curves were constructed to determine the cut-off values of HbA1c, HOMA-IR, HOMA-β, QUICKI and G/I ratio as predictors of AGT. Sensitivity, specificity, positive predictive value, negative predictive value and accuracy with 95% confidence intervals were calculated using a 2 × 2 table. Statistical significance was defined as a *p*-value of < 0.05. Analyses were performed using PASW Statistics, version 18.

## 3. Results

A total of 121 women diagnosed with PCOS were included. Their baseline characteristics are detailed in Table 1. The prevalence of AGT in this study was 24.8%, with 1.7% of the women having impaired fasting glucose, 18.2% having impaired glucose tolerance and 5.0% being diabetic. Table 2 compares the clinical and biochemical parameters of PCOS women with normal glucose tolerance and AGT. PCOS women with AGT exhibited significantly higher fasting glucose, fasting insulin, 2-h glucose, HbA1c, QUICKI, HOMA-IR, HOMA-β and G/I ratio values than those with normal glucose tolerance.

**Figure 1:**
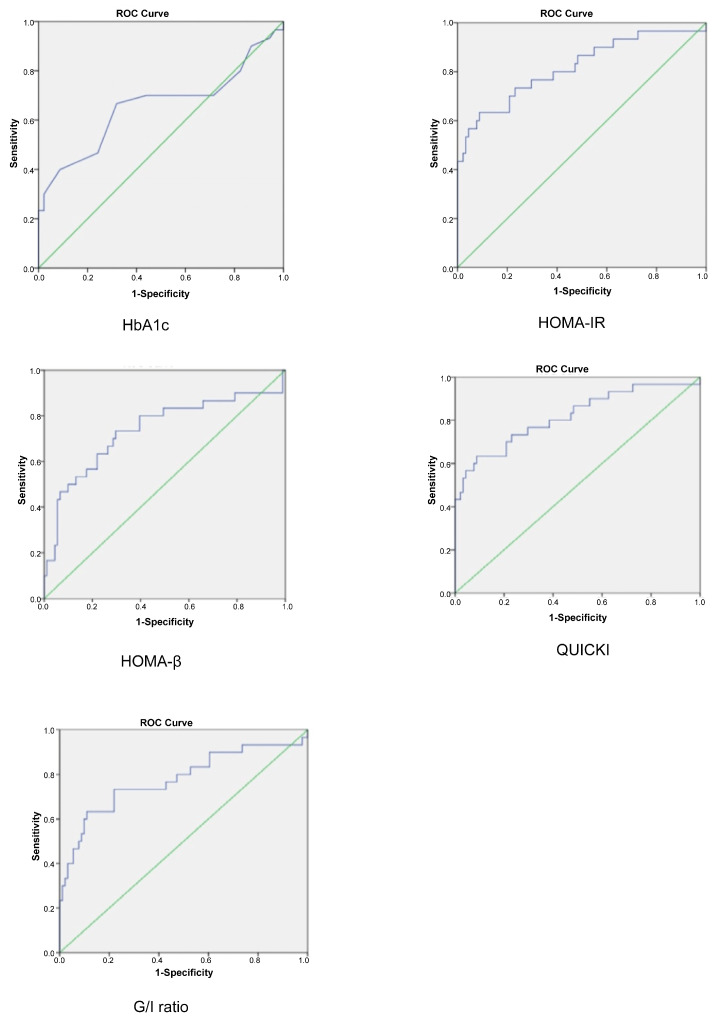
Receiver operating characteristic curves for serum markers in the detection of glucose intolerance in women with polycystic ovary syndrome. G/I ratio, fasting glucose-to-insulin ratio; HbA1c, haemoglobin A1c; HOMA-β, homeostasis model assessment of β-cell function; HOMA-IR, homeostasis model assessment of insulin resistance; QUICKI, quantitative insulin sensitivity check index.

The receiver operating characteristic analysis revealed areas under the curve (AUCs) of 0.656 for HbA1c, 0.817 for HOMA-IR, 0.737 for HOMA-β, 0.817 for QUICKI and 0.777 for the G/I ratio (shown in Figure 1). When compared with the 75 g OGTT results, HbA1c, HOMA-IR, HOMA-β, QUICKI and the G/I ratio exhibited variations in sensitivity, specificity, positive predictive value, negative predictive value and accuracy in detecting glucose intolerance among Thai women with PCOS (Table 3). After using multiple logistic regression, using BMI and age increased the AUC and accuracy for detecting AGT, especially with the GI ratio and BMI (AUC 0.791). However, the slight increase in AUC and accuracy was not significantly different from not using these factors.

## 4. Discussion

There have been limited reports on glucose intolerance in Asian women with PCOS. This study aimed to establish cut-off values of HbA1c and other serum markers to detect AGT in Thai women of reproductive age with PCOS. A key objective was to compare these serum markers as predictors of AGT, which has not been previously reported among Thai PCOS women.

The findings of this study indicate that HbA1c is not superior to other markers in detecting glucose intolerance. The prevalence of glucose intolerance in this study was 24.8%, comparable to values found by other Asian studies (20.0% and 22.5%) [13,23]. However, the prevalence of AGT in this study is lower than that reported in another Thai study by Charnvises et al. (42.9%) [24]. These differences could be attributed to population characteristic variations, as the current investigation participants were younger and leaner than those assessed by Charnvises and colleagues.

The prevalence of glucose intolerance in this study was higher than that in studies in Austria (14.1%) [25] and Turkey (16.3%) [22] but lower than that reported in the United States (35.3%) [26]. The Austrian and Turkish studies employed a 75 g oral OGTT for AGT detection, whereas the American study utilised HbA1c. Ethnicity, diet, lifestyle and testing methods may influence the prevalence of AGT. A previous study reported that PCOS women with glucose intolerance exhibited higher BMI, waist circumference, blood pressure, triglyceride levels, free testosterone levels, fasting glucose levels, HbA1c and insulin levels [27]. In particular, increased BMI was associated with glucose intolerance. Different methods for detecting AGT may contribute to variations in the prevalence of glucose intolerance.

The levels of HbA1c, fasting glucose and 2-hour glucose at baseline can serve as reliable predictors of type 2 DM. The PCOS guideline of 2018 recommends using the OGTT, fasting plasma glucose or HbA1c to assess glycaemic status in all women with PCOS [10]. However, only the OGTT can effectively detect glucose intolerance.

This study’s cut-off value of ≥5.4% for detecting glucose intolerance demonstrated acceptable sensitivity and specificity. This finding aligns with a similar study in Turkey, which employed a cut-off value of >5.35% [22]. Additionally, Lerchbaum et al., using a cut-off value of HbA1c > 5.7%, achieved a sensitivity of 25% and specificity of 100% in detecting glucose intolerance [25]. Conversely, Gooding et al. utilised an HbA1c cut-off value >5.7% and achieved a sensitivity of 60% and specificity of 69% [26]. There were differences in age between the present investigation and the work by Gooding and associates (26.8 vs. 16.6 years old). Moreover, the populations in the Gooding and Lerchbaum studies were predominantly Caucasian. These differences in age, ethnicity and AGT prevalence may contribute to variations in the cut-off points for detecting glucose intolerance [25,26,28].

Several factors can influence HbA1c levels, including abnormal haemoglobin, defective erythropoiesis, abnormal glycation, red blood cell destruction, underlying medical conditions and certain medications [29]. In this study, all PCOS women had no conditions that could affect HbA1c levels, thus eliminating potential confounding factors. The variations in the cut-off points for detecting glucose intolerance may be attributed to the characteristics of each ethnic population and lifestyle factors. Notably, the Asian populations studied tended to be younger and have a lower BMI than the Caucasian populations.

Previous Thai studies reported HbA1c reference intervals based on the Diabetes Control and Complications Trial/National Glycohemoglobin Standardization Program (DCCT/NGSP) or International Federation of Clinical Chemistry and Laboratory Medicine (IFCC) data. The HbA1c reference intervals were 5.47% (range: 4.79–6.15%) using DCCT/NGSP and 3.66% (range: 2.88–4.44%) using IFCC [30]. These intervals were consistent with another study utilising a 2.9–4.9% reference interval for HbA1c [31]. However, in the present investigation, a lower HbA1c threshold (DCCT/NGSP) was employed to identify abnormal HbA1c levels compared to the Thai population. This adjustment may be attributed to differences in the age of the study populations and the increased risk factors for metabolic syndrome observed in PCOS, necessitating a lower cut-off level for HbA1c detection.

This study revealed that HOMA-IR (cut-off point 2.3) and QUICKI (cut-off point 0.336) exhibited higher AUC values than other tests. The G/I ratio (cut-off point 6) also demonstrated the highest accuracy. These results suggest that HOMA-IR, QUICKI and the G/I ratio may serve as effective screening tests for evaluating glucose intolerance in PCOS women. However, these tests require calculated formulas, limiting their convenience for routine clinical use.

The cut-off value of ≥2.3 for HOMA-IR in this study aligns closely with findings from previous studies in Thailand (cut-off >2) [23] and China (cut-off >2.13) [13]. The higher prevalence of glucose intolerance observed in this study compared to previous Thai studies may account for the difference in the HOMA-IR cut-off level. Notably, the cut-off value of HOMA-IR in an Italian study was lower, which may be attributed to variations in ethnicity, age, central obesity and BMI [14].

In this study, a cut-off value of 0.336 or less for QUICKI was determined to detect glucose intolerance (sensitivity: 80%; specificity: 61.5%). These findings are consistent with those of Chen et al., who reported a cut-off value of 0.34 or less in Chinese PCOS women (sensitivity: 73.9%; specificity: 73.4%) [13].

The different cut-off values of the G/I ratio in this study and other studies [13,14,15,16] are likely due to the different characteristics of the study populations.

This study demonstrated that the AUC for HbA1c was 0.656, the lowest among all the markers assessed for detecting glucose intolerance. In other words, HbA1c showed lower accuracy for screening glucose intolerance in Thai PCOS women than other serum markers. Despite its convenience and lack of requirement for mathematical calculations, HbA1c was less reliable than other serum markers in this study.

While the G/I ratio offers a more precise assessment than other serum markers, the unavailability of basal glucose and insulin levels for all women with PCOS presents limitations. Therefore, this marker may not prove universally helpful, and the OGTT still remains necessary for all women with PCOS.

One strength of this study was the comprehensive evaluation of multiple methods and their comparative efficacy in detecting glucose intolerance in Thai urban women with PCOS. However, the sample was limited to patients from Siriraj Hospital, which limits the generalisability of the finding. In addition, these markers are not specific for assessing AGT in PCOS; however, we will try to determine if any can replace the 75 g OGTT. We still lack data comparing these markers to AGT in non-PCOS individuals. This study essentially introduces various markers representing the 75 g OGTT in an urban PCOS population. This can serve as a model for future research involving data collection from multiple locations.

## 5. Conclusions

In conclusion, HbA1c may not be a reliable indicator for detecting glucose intolerance in Thai PCOS women compared with other tests. Although HbA1c is easy to obtain, convenient and quick to test, HOMA-IR, QUICKI and the G/I ratio are more accurate.

## Figures and Tables

**Table 1:** Baseline characteristic of 121 women with polycystic ovary syndrome.

Parameters	Mean ± SD, Median (P25, P75) or *n* (%)
Age (yr)	26.2 ± 5.3
Parity	
Yes	9 (24.8)
No	112 (75.2)
BMI	25.5 ± 6.9
Underweight	12 (9.9)
Normal	39 (32.2)
Overweight	17 (14.0)
Obese	53 (43.8)
Waist circumference (cm)	83.3 ± 17.9
Presence of acanthosis nigricans	23 (19.0)
MFGs	4 (3, 5)
Haemoglobin (g/dL)	13.1 ± 1.2
Total testosterone (ng/mL)	0.4 ± 0.3
Free testosterone (ng/dL)	0.7 ± 0.5
Fasting glucose (mg/dL)	82.5 ± 24.2
Fasting insulin (µU/mL)	10.91 (10.93, 19.20)
2-hour glucose (mg/dL)	124.6 ± 54
HbA1c (%)	5.5 ± 0.9
QUICKI	0.3 ± 0.1
HOMA-IR	2.29 (1.34, 4.01)
HOMA-β	197.33 (128.46, 321.50)
G/I ratio	7.76 (4.40, 11.94)

BMI, body mass index; G/I ratio, fasting glucose-to-insulin ratio; HbA1c, haemoglobin A1c; HOMA-β, homeostasis model assessment of β-cell function; HOMA-IR, homeostasis model assessment of insulin resistance; MFGs, modified Ferriman–Gallwey score; QUICKI, quantitative insulin sensitivity check index; SD, standard deviation.

**Table 2:** Clinical and biochemical parameters of women with polycystic ovary syndrome stratified by normal and abnormal glucose tolerance test results using 75-gram oral glucose tolerance test.

Parameters	Normal Glucose Tolerance Test (*n* = 91)	Abnormal Glucose Tolerance Test (*n* = 30)	*p*
Age (yr)	25.6 ± 5.3	27.8 ± 5.2	0.05
Parity			
Yes	6 (6.6)	3 (10.0)	0.681
No	85 (93.4)	27 (90.0)	
BMI	23.8 ± 5.7	30.6 ± 7.9	<0.01
Underweight	10 (11.0)	2 (6.7)	
Normal	37 (40.7)	2 (6.7)	
Overweight	14 (15.4)	3 (10.0)	
Obese	30 (33.0)	23 (76.7)	
Waist circumference (cm)	79.7 ± 12.2	94.0 ± 17.3	<0.01
Acanthosis nigricans	10 (11.0)	13 (43.3)	<0.01
MFGs	4 (2, 5)	4 (3, 5)	0.700
Haemoglobin (g/dL)	12.9 ± 1.2	13.5 ± 1.4	0.013
Total testosterone (ng/mL)	0.4 ± 0.2	0.6 ± 0.6	0.148
Free testosterone (ng/dL)	0.6 ± 0.4	1.0 ± 0.8	0.007
Fasting glucose (mg/dL)	82.3 ± 6.9	103.4 ± 43.8	0.014
Insulin (µU/mL)	9.81 (6.06, 14.29)	27.39 (12.86, 39.37)	<0.01
2-h glucose (mg/dL)	102.8 ± 19.1	190.5 ± 70.4	<0.01
HbA1c (%)	5.3 ± 0.3	6.0 ± 16	0.022
QUICKI	0.35 ± 0.04	0.31 ± 0.05	<0.01
HOMA-IR	2 (1.21, 2.95)	6.52 (2.64, 9.59)	<0.01
HOMA-β	180.57 (123.87, 255.29)	351.31 (209.26, 493.78)	<0.01
GI ratio	8.28 (6, 13.37)	3.83 (2.43, 7.91)	<0.01

Data are presented as mean ± SD, median (P25, P75) and *n* (%). BMI, body mass index; G/I ratio, fasting glucose-to-insulin ratio; HbA1c, haemoglobin A1c; HOMA-β, homeostasis model assessment of β-cell function; HOMA-IR, homeostasis model assessment of insulin resistance; MFGs, modified Ferriman–Gallwey score; QUICKI, quantitative insulin sensitivity check index; SD, standard deviation.

**Table 3:** Diagnostic test values of serum markers for the detection of glucose intolerance in women with polycystic ovary syndrome.

Test	AUC	Cut-Off Point	Sensitivity (%)	Specificity (%)	PPV (%)	NPV (%)	Accuracy (%)
HbA1c	0.656	5.4	70.0	56.0	34.4	85.0	59.5
HOMA-IR	0.817	2.3	80.0	60.4	40.0	90.2	65.0
HOMA-β	0.737	200.0	80.0	60.4	40.0	90.2	65.3
QUICKI	0.817	0.336	80.0	61.5	40.7	90.3	66.1
G/I ratio	0.777	6.0	73.3	74.7	48.9	89.5	74.4

AUC, area under the curve; G/I ratio, fasting glucose-to-insulin ratio; HbA1c, haemoglobin A1c; HOMA-β; homeostasis model assessment of β-cell function; HOMA-IR, homeostasis model assessment of insulin resistance; NPV, negative predictive value; PPV, positive predictive value; QUICKI, quantitative insulin sensitivity check index.

## Data Availability

The original contributions presented in this study are included in the article. Further inquiries can be directed to the corresponding author.
